# Reproductive performance following hysteroscopic treatment of intrauterine adhesions: single surgeon data

**DOI:** 10.52054/FVVO.14.1.005

**Published:** 2022-04-03

**Authors:** L.S. Direk, M Salman, A Alchami, E Saridogan

**Affiliations:** University College London, Elizabeth Garrett Anderson Institute for Women’s Health; University College London Hospital, Reproductive Medicine Unit.

**Keywords:** Intrauterine adhesions, Asherman syndrome, hysteroscopic treatment, reproductive outcome

## Abstract

**Background:**

Intrauterine adhesions can negatively affect reproductive outcomes by causing infertility, miscarriage and preterm birth in women. Hysteroscopic surgery is now widely accepted as the treatment of choice in symptomatic women to restore reproductive function.

**Objectives:**

To analyse the patient characteristics and long-term reproductive outcomes of women who received treatment for intrauterine adhesions under the care of a single surgeon.

**Materials and Methods:**

In this retrospective analysis, all women who underwent hysteroscopic surgery for intrauterine adhesions under the care of the same surgeon between January 2001 and December 2019 were identified and their data were evaluated. Relevant demographic, diagnostic and reproductive outcome data was procured from patient notes. Referring doctors and patients were contacted to obtain missing information.

**Main outcome measures:**

Live birth and miscarriage rates.

**Results:**

126 women were treated for intrauterine adhesions. Of those women who were trying to conceive, 71.4% (65/91) achieved pregnancy, 58.2% (53/91) had live births and 13.2% (12/91) had miscarriages. No statistically significant difference was found in the live birth rates when data was analysed in subgroups based on age, reason for referral/aetiology and severity of pathology.

**Conclusions:**

Hysteroscopic surgery leads to live birth in the majority of women with intrauterine adhesions. The lack of statistically significant difference in live birth rates across subgroups, including advanced age and severe pathology, suggests that surgery in all women wanting to conceive can be justified.

**What is new?:**

Hysteroscopic treatment can lead to successful outcomes even in the presence of severe adhesions and in older women with appropriate treatment.

## Introduction

Since the end of the nineteenth century, intrauterine adhesions (IUAs) have been acknowledged to be a consequence of uterine trauma (Fritsch, 1894). In particular, trauma to the basal layer of the endometrium, often subsequent to surgery and inflammation, can lead to formation of IUAs (Devi Wold et al., 2006; Torres-De La Roche et al., 2019). The true incidence of IUAs is difficult to identify, due to an unknown number of asymptomatic cases who do not present to the health services. The number of cases reported have increased in more recent years, possibly due to the growing number of complicated pregnancies and uterine surgeries, combined with better diagnosis (Deans and Abbott, 2010).

IUAs linked to pregnancy are the most common, with 21.5% to 40% of those who undergo postpartum uterine instrumentation developing IUAs (Deans and Abbott, 2010). Curettage plays an important role in the development of IUAs (Asherman, 1950), particularly when repeated multiple times (Friedler et al., 1993). A study of 1,856 women with IUAs showed that 89% of these women had previously experienced pregnancy termination, miscarriage or postpartum haemorrhage which was treated with curettage (Schenker and Margalioth, 1982).

IUA development can also be unrelated to pregnancy, with resectoscopic surgery playing an important role. Although risk of IUA formation after hysteroscopic myomectomy was reported as approximately 10% (Hallez, 1995), another study reported frequency of IUAs after resection of one myoma as 31.3%, multiple myomas as 45.5% and uterine septa as 6.7% (Taskin et al., 2000). In particular, there is an increased risk of IUA formation when multiple fibroids are resected during the same procedure from opposing walls of the uterus (Saridogan, 2016). Risk of IUA formation after abdominal surgery involving the uterus varies from approximately 2% following a caesarean section, to 1.3-25.51% following abdominal myomectomy (Capmas et al., 2018; Schenker and Margalioth, 1982).

Although IUAs can be asymptomatic, common symptoms include menstrual disturbance, pelvic pain, subfertility and miscarriage (Deans and Abbott, 2010; Schenker and Margalioth, 1982).

Menstrual disturbance is a very common presentation of IUAs, mostly presenting as amenorrhoea or hypomenorrhoea, although in a small proportion of cases there is menorrhagia or normal menses (Schenker and Margalioth, 1982).

If left untreated, approximately 40% of pregnancies in women with IUAs end in miscarriage and 23% in preterm delivery (Schenker and Margalioth, 1982). Recurrent miscarriage may be linked to suboptimal placentation and to the reduction in the size of the uterine cavity, subsequently causing pregnancy loss (Devi Wold et al., 2006). Subfertility has been theorised to be due to sperm obstruction, problems with embryo migration, or implantation difficulty due to endometrial insufficiency (Deans and Abbott, 2010). Vascular damage to the endometrium has been identified as a possible factor leading to adverse pregnancy outcome (Polishuk et al., 1977).

Hysteroscopic division of IUAs is the widely accepted treatment choice in symptomatic women. The aim of treatment is to restore the uterine cavity, allow communication between the cervix and tubal ostia, facilitate endometrial development for successful implantation of the embryo and development of pregnancy (Abbott et al., 2017). However, the efficacy of treatment, reproductive performance and prognostic factors have not been fully elucidated (Grimbizis et al., 2021). Many publications combine data from different centres and multiple surgeons who may have different techniques and variable levels of experience. We therefore aimed to analyse the patient characteristics, surgical approaches and long-term reproductive outcomes of women who received treatment for IUAs under the care of a single surgeon.

## Methodology

### Study Design and Population

This was a retrospective analysis of women who underwent surgical treatment for IUAs between January 2001 and December 2019 under the care of a single surgeon (ES).

Patients were identified from hospital operating theatre records and consultant diaries. Data were collected on patient demographics, presenting symptoms, pre-operative diagnostic methods and their findings, operative details and complications and post-operative outcomes.

For each patient, relevant data was procured from their electronic or paper records, or clinic letters. Findings were entered into a password-protected Excel spreadsheet (Microsoft Corporation, Seattle, WA, USA).

All procedures were performed by a single surgeon (ES), using a similar technique under general anaesthesia as a day procedure. Hysteroscopic procedures were performed using a minihysteroscope (Alphascope, Johnson and Johnson, or 4.2 mm Bettocchi operating hysteroscope, Karl Storz) with inflow, outflow and operating 5 or 7 French operating channels. An ultrasound guidance was utilised for the majority of patients. The severity of adhesions were classified according to the American Fertility Society Classification of Intrauterine Adhesions system, based on hysteroscopic findings and the menstrual pattern (The American Fertility Society, 1988).

IUAs were divided using hysteroscopic scissors and use of energy was avoided. The aim was to achieve a regular cavity and reach tubal ostia on both sides. When tubal ostia could not be found or were stenosed, achieving a symmetrical cavity was aimed. Once a regular cavity was achieved, either hyaluronic acid gel or one of two copper intrauterine contraceptive devices (IUCDs) of different shapes were inserted into the cavity for secondary adhesion prevention. Patients were then given oral oestradiol tablets of 2-4 mg daily for 1-3 months. A repeat hysteroscopy was performed routinely to check the cavity and treat any persistent/recurrent adhesions, unless the adhesions were minimal/ mild. IUCDs were removed once a good outcome was achieved, as confirmed at repeat hysteroscopy. Repeat hysteroscopies were usually performed as outpatient procedures, unless it was anticipated that further treatment would be required.

### Statistical Analysis

Statistical analysis was performed using Excel (Microsoft Corporation, Seattle, WA, USA).

Proportions are expressed as percentages with 95% confidence intervals (CIs) and subgroup data has been analysed using the Chi-squared test in order to give a P-value to ascertain whether the data is of statistical significance.

### Ethical Approval

The study was assessed by the NHS Health Research Authority ‘Defining Research’ decision tool and full ethical review by an NHS Research Ethics Committee or NHS/Health and Social Care Research and Development office was not required as this study was considered a service evaluation (www.hra-decisiontools.org.uk/research/).

## Results

### Study population:

During the study period, 126 women were treated for IUAs, with a mean age of 38.2 years (range 25 – 52 years). History of previous miscarriage managed with evacuation of retained products (ERPC), was the most common feature, followed by postpartum ERPC and open myomectomy. There was no obvious identifiable cause of IUAs in 14 of 126 cases (Table I).

**Table I:** Medical/obstetric history upon presentation of patients who received treatment for IUAs.

History (N=126)	Number	%	95% CI
Postpartum – ERPC	20	15.9	9.5-22.3
Postpartum infection	3	2.4	0-5.0
Post C-section	14	11.1	5.6-16.6
Miscarriage - ERPC^a.b.c^	34	27.0	19.2-34.7
Miscarriage - unknown management	3	2.4	0-5.0
Termination of pregnancy	10	7.9	3.2-12.7
Open myomectomy^d^	16	12.7	6.9-18.5
Transcervical resection of fibroids	5	4.0	0.6-7.4
Uterine artery embolisation	1	0.8	0-2.3
Polypectomy	4	3.2	0.1-6.2
Uterine septum division	2	1.6	0-3.8
No identifiable cause in history	14	11.1	5.6-16.6
Total	126	100.0	

^a^one with post C-section; ^b^one with uterine artery embolisation; ^c^ one with open myomectomy; ^d^ one with transcervical resection of fibroids

Initial diagnosis was made using hysteroscopy in 53 of 126 (42.1%; 95% CI, 33.4-50.7) cases, ultrasound (with or without saline infusion) in 66 of 126 (52.4%; 95% CI, 43.7-61.1) cases and hysterosalpingography in 6 of 126 (4.8%; 95% CI, 1.0-8.5) cases. In one case (0.8%; 95% CI, 0-2.3), a combination of ultrasound and hysterosalpingography was used. All diagnoses were confirmed at hysteroscopy when adhesions were treated.

**Figure 1:**
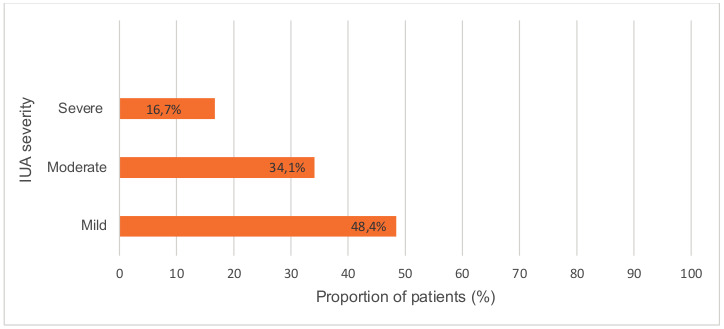
Stage of IUAs based on American Fertility Society Classification of Intrauterine Adhesions system in the study population.

61 of 126 (48.4%; 95% CI, 39.7-57.1) patients had mild IUAs, with concomitant retained products of conception (RPOC) in one of these patients, and concomitant submucosal fibroids in another. 43 of 126 (34.1%; 95% CI, 25.8-42.4) of women had moderate IUAs and 21 of 126 (16.7%; 95% CI, 10.2-23.2) had severe IUAs (Figure 1). In one case (0.8%; 95% CI, 0-2.3), the severity of IUAs was not clear in available records.

### Post-treatment reproductive outcome:

Following treatment, 25 of 126 (19.8%; 95% CI, 12.9-26.8) women were lost to follow-up, 5 of 126 (4.0%; 95% 0.6-7.4) women are awaiting IVF (in vitro fertilisation) treatment, 1 of 126 (0.8%; 95% 0.1-2.3) is not trying for pregnancy yet and 4 of 126 (3.2%; 95% 0.1-6.2) decided against fertility treatment. The remaining 91 of 126 patients have been analysed for reproductive outcome.

Overall, of those women trying to conceive, 65 of 91 (71.4%; 95% CI, 62.1-80.7) achieved pregnancy, 12 of 91 (13.2%; 95% CI, 6.2-20.1) had miscarriages and 53 of 91 (58.2%; 95% CI, 48.1-68.4) had live births (total 55 deliveries, 2 sets of twins).

27 of 91 (29.7%; 95% CI, 20.3-39.1) patients conceived spontaneously. Of these women, 23 of 27 (85.2%; 95% CI, 71.8-98.6) had live births and 4 of 27 (14.8%; 95% CI, 1.4-28.2) had miscarriages.

38 of 91 (41.8%; 95% CI, 31.6-51.9) patients conceived with IVF. Of these women, 30 of 38 (78.9%; 95% CI, 66.0-91.9) had live births and 8 of 38 (21.1%; 95% CI, 8.1-34.0) had miscarriages.

16 of the total 91 women (17.6%; 95% CI, 9.8- 25.4) received IVF treatment but failed to conceive. 2 of 91 (2.2%; 95% CI, 0-5.2) women opted for surrogacy.

The post-treatment reproductive outcomes are summarised in Table II.

**Table II:** Post-treatment reproductive outcomes of patients known to be trying for pregnancy.

Outcome (N=91)	Number	%
Pregnant spontaneous	27	29.7
Miscarriage	4	14.8
Live birth	23	85.2
Pregnant with IVF	38	41.8
Miscarriage	8	21.1
Live birth	30	78.9
Did not conceive	26	28.6
Total	91	100.0

### Live birth rates in subgroups:

Data were analysed in subgroups based on age, possible underlying aetiology, severity of IUAs and number of hysteroscopies required for treatment.

**Figure 2:**
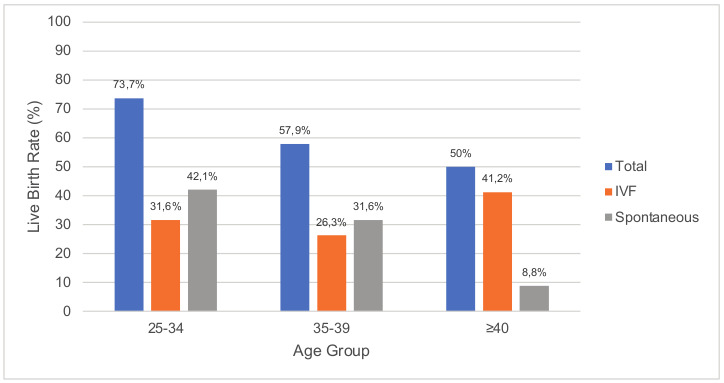
The live birth rates in different age groups following treatment for IUAs.

Live births occurred in 17 of 34 (50.0%; 95% CI, 33.2-66.8) cases in the ≥40 years age group, in 22 of 38 (57.9%; 95% CI, 42.2-73.6) cases in the 35-39 age group and in 14 of 19 (73.7%; 95% CI, 53.9-93.5) cases in the 25-34 age group. There was a trend of higher pregnancy rates in younger women but the differences in live birth rates across the age groups were not statistically significant (p=0.24). The live birth rates in different age groups are shown in Figure 2.

The live birth rates in the different aetiological categories are summarised in Table III. The differences in live birth rates across the categories were not statistically significant (p=0.73).

**Table III:** The live birth rates based on the relevant histories of patients.

History (N=91)	Number trying	Live birth	% live birth (95% CI)
Postpartum - evacuation of retained products	17	12	70.6 (48.9-92.2)
Postpartum infection	2	2	100
Post C-section	12	7	58.3 (30.4-86.2)
Miscarriage - evacuation of retained products	24	14	58.3 (38.6-78.1)
Miscarriage - unknown management	2	1	50.0 (0-100)
Termination of pregnancy	7	2	28.6 (0-62.0)
Open myomectomy	11	7	63.6 (35.2-92.1)
Transcervical resection of fibroids	2	1	50.0 (0-100)
Uterine artery embolisation	1	0	0
Polypectomy	4	2	50 (1.0-99.0)
Uterine septum division	1	1	100
No identifiable cause in history	8	4	50.0 (15.4-84.6)

**Figure 3:**
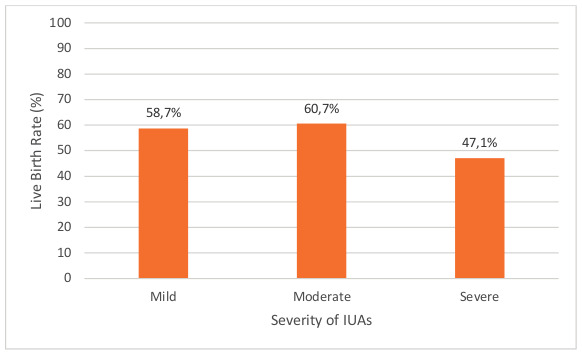
The live birth rates for different severities of IUAs following treatment.

There was a result of live birth in 27 of 46 (58.7%; 95% CI, 44.5-72.9) cases in women with mild adhesions, in 17 of 28 (60.7%; 95% CI, 42.6- 78.8) cases in women with moderate adhesions and in 8 of 17 (47.1%; 95% CI, 23.3-70.8) cases in women with severe adhesions (Figure 3). The differences in live birth rates across the different severities of intrauterine adhesions were not statistically significant (p=0.64).

The live birth rates were 23 out of 37 (62.2%; 95% CI, 46.5-77.8) in women who required one hysteroscopic procedure, 27 out of 49 (55.1%; 95% CI, 41.2-69.0) after 2-3 hysteroscopies and 3 out of 5 (60.0%; 95% CI, 17.1-100) after 4 or more hysteroscopies (p=0.80). The maximum number of hysteroscopies performed in one case was 6.

## Discussion

This retrospective evaluation of hysteroscopic treatment of IUAs in the practice of a single surgeon showed that 71% of women conceived either spontaneously or following IVF/ICSI treatment and over 58% had live births. A significant proportion of women required multiple procedures, some requiring 4 or more procedures. The pregnancy rates were similar in women with mild, moderate or severe adhesion categories and age of the women did not seem to have a significant impact on success rates, although there was a trend towards better success rates in younger women.

The most common histories upon presentation were miscarriage managed with ERPC and postpartum ERPC (27% and 15.9% respectively). In total, 66.7% of patients presented with pregnancy related histories. This is consistent with the published literature which suggests that IUA formation is most commonly related to pregnancy, and more specifically postpartum uterine instrumentation, with 21.5% to 40% of these women developing IUAs (Deans and Abbott, 2010). Postpartum placental retention is a particularly high-risk background for IUA.

Retained placenta usually causes inflammatory reaction or infection in the uterine cavity. A surgical procedure is required to remove the placental tissue, which probably adds to further trauma to the intrauterine decidua. Furthermore, the hypo-oestrogenic post-partum environment may facilitate adhesion formation, as oestrogens are required for regeneration of the endometrium. Repeat surgical procedures following miscarriages cause IUAs in a similar way, except the presence of hypo-oestrogenaemia. It is known that the number of procedures required for the treatment of miscarriages is related to the risk of IUAs (Hooker et al., 2014).

In our series, open myomectomy was the third most common history upon presentation (12.7%), which is more supportive of the literature reporting rates of IUA development of up to 25.5% (Capmas et al., 2018) after this procedure, rather than studies reporting risk as low as 1.3% (Schenker and Margalioth, 1982). IUAs are probably related to the opening of the cavity during myomectomy (Şükür and Saridogan, 2021). It is also quite likely that the risk of adhesions increases with the number and size of cavity breaches. Another reason for IUAs following myomectomy is that the sutures which are used to repair the myoma bed may inadvertently go through the uterine cavity and stick the opposing endometrial surfaces together. Awareness of risk of IUAs following an abdominal myomectomy is probably an important step in prevention; when possibility of IUAs is anticipated (cavity breach or myometrial sutures), early hysteroscopic assessment of the uterine cavity in the postoperative period may be beneficial (Şükür and Saridogan, 2021).

14% of patients had no identifiable cause of IUAs in their history. These patients may have had pregnancies in the past and felt unable to mention these during their consultations.

Ultrasound was the most common method of diagnosis for IUAs overall in our practice. Despite hysteroscopy being considered the gold standard for diagnosis (March et al., 1978), this method is more invasive when compared to ultrasound, which is often preferred. Ultrasound is cheap, accessible and non-invasive, making it an ideal diagnostic method in experienced hands (Amin et al., 2015). Ultrasound can also be used to monitor women who are at risk of developing IUAs following a complicated pregnancy (Amin et al., 2015).

The overall 71.4% pregnancy rate is somewhat higher than the average success in comparison to an analysis of 36 studies up to 2008 reporting on reproductive outcome in women who underwent the same procedure, which shows an approximate pregnancy rate of 63% (Deans and Abbott, 2010). Our results are closer to a more recent publication which reported a pregnancy rate of 79% (Deans et al., 2018). The higher pregnancy rate in our series could be explained by the fact that all of the procedures were undertaken by a single experienced surgeon with the use of a standardised technique with miniaturised instruments. Live birth rate (of those who conceived) was reported as 75% in the analysis of 36 studies (Deans and Abbott, 2010), which included a study reporting a live birth rate as high as 93% (Feng et al., 1999). In our study, live birth rate (of those who conceived) was 82% (53/65). The differences in pregnancy and live birth rates between studies could be due to the variations in patient mix, IUA severity and age of patients which introduces heterogeneity. Another factor which would have an impact on reproductive outcomes is the source of referral of the patients. A large majority of patients in our study were referred from fertility clinics, making it possible that they had additional factors affecting their fertility other than adhesions. In fact, a large proportion (38/91 or 41.8% following IVF as opposed to 27/91 or 29.7% spontaneous) of our patients conceived following IVF and this indicates that their infertility is more likely to be due to multiple factors. The high pregnancy rate despite these additional factors is very encouraging and justifies hysteroscopic treatment in women experiencing infertility.

Interestingly, those who conceived spontaneously had a higher proportion of successful live births (85.2% and 78.9% respectively) as well as a lower proportion of miscarriages (14.8% and 21.1% respectively).

### Live birth rates in subgroups:

Live birth rate was highest in women aged 25 to 34 years (73.7%) and lowest in women aged ≥40 years (50.0%), although not statistically significant, possibly due to the small sample sizes. The data however suggest that even older women have a chance at having a successful live birth following treatment, albeit some have used donor eggs to conceive. From those with known outcomes, all women aged ≥45 years in this study underwent or are waiting to undergo IVF in order to conceive, and would presumably make use of donor gametes, therefore eliminating the issue of low ovarian reserve and fertility potential.

While the live birth rates with IVF and spontaneous conception are very similar in women aged ≤39 years, live births following IVF were more likely in women aged ≥40 years, as expected (41.2% and 8.8% respectively).

There was a trend towards higher live birth rates in women who had a history of postpartum ERPC (70.6%), followed by open myomectomy (63.6%). These figures are similar to what was reported in a recent article by Hanstede et al. (2021) who reported live birth rate of 67% following surgical treatment of Asherman syndrome. Overall, the differences were not statistically significant across the ten categories. When grouped into pregnancy related aetiology and non-pregnancy related aetiology, the live birth rates are 59.4% (38/64) and 57.9% (11/19) respectively.

Interestingly, the live birth rates in women with mild, moderate and severe IUAs were similar. This is unusual, as one would expect a negative correlation between IUA severity and term pregnancy rate (Deans et al., 2018). However, our data show good outcomes even with severe adhesions, despite reports in published literature, suggesting that treatment is worthwhile and can produce favourable results regardless of the severity of the IUAs. One study reported a live birth rate of 61% in women who had moderate or severe IUAs (Xiao et al., 2014), compared to 55.6% (25/45) in our study. It is however difficult to compare the two values, as the aforementioned study calculated the live birth rate as a proportion of those who conceived, while in our study it is calculated as a proportion of all those who were trying for pregnancy.

Published literature has indicated that hysteroscopic adhesiolysis can result in normal cavity restoration in one to three operations in up to 95% of cases (Hanstede et al., 2015), which is comparable to the 96% of cases successfully treated in one to three operations in this study. Recurrence of IUAs may be linked to the degree of adhesions, with a recurrence rate of 20% to 62.5% in severe adhesions (Yu et al., 2008), therefore a negative correlation between number of operations needed to treat and live birth rate might be expected. Conversely, in our study those who underwent four or more hysteroscopies still showed high live birth rates (60%), although the numbers were small. Nevertheless, this is a good indication that even women who undergo multiple surgeries to treat IUAs have a chance at having successful pregnancies, and it shows the value of having follow-up hysteroscopies to monitor and treat recurrent adhesions. It is important to note that in some cases repeat hysteroscopies were diagnostic only in our study, as examination did not show any recurrence or persistence of IUAs and therefore operation was not required.

One of the strengths of our study is that this is a single centre and single surgeon series in which the same surgical technique was used consistently, eliminating the variability seen in other published studies. Additionally, long term follow-up allowed identification of reproductive outcomes in the majority (~80%) of patients. Reproductive outcomes were available from patients with relatively complex backgrounds, including many who were referred from fertility clinics with a history of multifactorial infertility.

One of the limitations is that the retrospective nature of this study resulted in some missing data, however despite this, it was possible to identify the reproductive outcomes in the majority of cases. Secondly, due to the nature of the study, we were not able to compare the results to a control group who would be managed expectantly. This would have provided a much clearer picture of how effective the treatments were. However, many would consider using a control group who would be offered expectant management unethical, especially for IUAs. A larger sample size would have been valuable when calculating reproductive outcome rates in subgroups and statistical significance of results.

## Conclusion

Overall, encouraging reproductive outcomes were observed for women undergoing hysteroscopic surgery for IUAs in a patient population with a wide range of background characteristics including those with additional causes of infertility. There was no statistically significant difference in live birth rates when data were analysed in subgroups based on age, aetiology/reason for referral, and severity of uterine pathology, suggesting that surgery can be justified in all women seeking to conceive, including those presenting at an older age or with a more severe form of the condition. Our study results present evidence that hysteroscopic surgery for IUAs can have a valuable impact on successful reproductive outcome.
